# Newly diagnosed type 2 diabetes – does New Zealand General Practice adequately prepare patients to self-manage their Condition?

**DOI:** 10.1186/s12875-023-02118-1

**Published:** 2023-08-21

**Authors:** Lynne Chepulis, Jessie Mellsop-Kupe, Suzanne Moorhouse, Rawiri Keenan, Kimberley Norman, Ryan Paul

**Affiliations:** 1 Waikato Medical Research Centre, Te Huataki Waiora School of Health, University of Waikato, Private Bag 3105, Hamilton, New Zealand; 2 Te Whatu Ora Health New Zealand, Hamilton, New Zealand; 3 Hauraki Primary Healthcare Organisation, Hamilton, New Zealand

**Keywords:** Type 2 diabetes, New diagnosis, General practice

## Abstract

**Background:**

Type 2 diabetes mellitus (T2D) is predominantly managed in primary care, and patients need to be provided with appropriate knowledge and education to understand how to best self-manage their condition. For optimal T2D self-management, primary care teams should share this information from the time of diagnosis. Little is currently known about how and when these resources are being provided to patients with T2D in New Zealand.

**Methods:**

An online survey was carried out between Oct 2022 and Feb 2023. Patients diagnosed with T2D after Jan 2020 were invited to participate, with recruitment occurring via primary care and social media. Questions included information about demographics, diagnosis, provision of education resources and/or referral services as well as about current diabetes management. All responses were analysed with chi square tests. Free-text comments were summarised only.

**Results:**

A total of 203 participants from across New Zealand completed the survey, but 18 were excluded due to being diagnosed more than 3 years ago, or self-reporting with type 1 diabetes rather than T2D. Nearly three quarters (70.7%) of participants reported that they were given appropriate resources to understand and manage their T2D, though half of these would have like more information. Overall, family and friends, self-led research and healthcare-provided education were equally useful, though this differed by ethnic groups. Similarly, approx. 70% of patients thought that medications had been well explained. Free text comments suggested a need for more targeted information around food choice and insulin use as well as a need for empathy and appropriate language from healthcare providers.

**Conclusions:**

Primary care appears to be providing most newly diagnosed patients with appropriate resources to understand and manage their T2D, but there is room for improvement with up to a third of participants not understanding how to manage foods, medication and lifestyle choices to optimise health outcomes. Further work is required to address this gap and should include the use of culturally-appropriate materials to meet the multi-ethnic population needs.

**Supplementary Information:**

The online version contains supplementary material available at 10.1186/s12875-023-02118-1.

## Introduction

Type 2 diabetes mellitus (T2D) is an increasingly prevalent health issue, both in New Zealand [1] and worldwide [2]. Importantly, it is also a driver of health inequity, particularly for Indigenous populations [3, 4]. In New Zealand, Indigenous Māori are diagnosed with T2D at twice the rate of non-Māori, and their rate of diabetes-related complications are more than five-times higher [5]. Whilst the reasons behind this are complex and rooted in racism and colonisation, [6] improving T2D health outcomes is essential to reduce and eventually eliminate health inequity, thus ensuring optimal disease management and quality of life for all patients.

T2D is generally diagnosed and managed in primary care, which in New Zealand can include either a traditional general practice team or a Māori health provider (primary care services delivered within a Māori-defined framework) [7]. Traditionally, diagnosis has tended to occur in mid-life, though more recently increasing numbers of patients of a younger age (< 40 years) are being diagnosed [2]. This creates a number of serious health challenges as ‘young-onset’ T2D have been shown to have a significantly higher risk of mortality and cardiovascular disease [8, 9].

Importantly, regardless of the age at diagnosis, T2D can be well managed with appropriate input from both the patient and the clinical team. Lifestyle changes are the gold standard of management at all stages of T2D, but education and advice needs to be provided in a timely and informative way by the primary care team [10]. In addition, patients should be aware of the need for ongoing and regular reviews of blood glucose levels and cardiovascular/renal risk factors. Medication use also needs to be optimised, as required, to reduce glycated haemoglobin (HbA1c) levels and to minimise the likelihood and/or progression of disease complications and mortality [10].

Alongside clinical input there is also a need for patients to self-manage their T2D. To optimise this, they must be well equipped with knowledge about how to effectively control their glucose levels from the time of diagnosis. Understanding how diabetes effects the body and how medications work are vital for effective management, [11] and guidelines recommend that all diabetes patients should be provided with relevant diabetes education to support disease self-management [10, 12]. Indeed, previous literature has reported that timely self-management education not only improves clinical measures such as HbA1c, [13] it also reduces all-cause mortality [11] and improves patient quality of life [14]. In particular, information and education provided at or from the time of diagnosis has shown to be highly effective at supporting diabetes self-management [15, 16].

In New Zealand, patients with T2D are usually diagnosed through screening (e.g. as a part of the cardiovascular risk assessment offered to all adults) or via presentation of symptoms to a general practitioner (GP) or other primary care practitioner. Provision of care often differs greatly across practices, but many are then offered an appointment with a GP or nurse, allowing time to undertake a full clinical diabetes review and to provide the opportunity for the patient and/or wider family to question and discuss their disease. The primary care team can also refer the patient onto additional services (e.g. dietician or psychological services, though these services are limited) and/or provide diabetes-specific resources such as those available from Diabetes New Zealand, a charitable trust developed with the sole of aim of supporting patients to manage diabetes.

However, despite the presence of these services, primary care data suggests that many patients with T2D are not meeting clinical targets or achieving optimal health outcomes [17]. Studies have shown that this may be because interactions with clinical staff can actually present as a barrier to ongoing healthcare, [18, 19] particularly if this is not delivered in a culturally appropriate manner [20]. It has also been identified that clinicians themselves sometimes do not feel adequately trained to deliver specific diabetes information, [21, 22] and that appropriate resources can be either unavailable or inappropriate depending on the age and/or ethnicity of the patient [23]. Online content, for example is often US-centric [24] and unregulated health information such as ‘health experts’ on social media platforms can cause confusion for patients [25].

Thus, despite the fact that the literature strongly supports the use of patient-level T2D education, [10, 12] including programs and resources that are culturally appropriate, [26] we suggest that activity varies widely across primary care, and that there is a gap in the New Zealand healthcare system where some patients with newly diagnosed T2D are not be being provided with timely (i.e. around the time of diagnosis) and/or understandable information. Thus, the aim of this study was to survey the self-reported understanding of T2D in a cohort of newly diagnosed patients and to identify the information they were provided with, including the sources that patients were utilising to find information.

## Methods

Following ethics approval from the University of Waikato Health Ethics Committee (HREC(Health)2020#24) an online survey was collectively developed by the research team to assess different aspects of patient experiences of managing T2D in the 12–36 months following diagnosis. This was initially piloted on a small group of people with known T2D (n = 6) to ensure readability and appropriate question interpretation.

The survey was made available via Survey Monkey between 01 and 2022 and 28 February 2023. Initially, patients enrolled with a regional primary healthcare organisation (of which approximately half identify as Māori) who had a diagnosis of T2D recorded in their clinical records at or after Jan 01 2020 were invited via text to opt in to the survey. Secondly, information about the survey and the survey link were posted on a number of Diabetes New Zealand Facebook groups and patients self-identifying with a diagnosis of T2D after Jan 01 2020 we invited to participate. These links remained active for the duration of the study period, and were reposted and shared to seek further responses. All responses were anonymous (thus not validated against clinical records), and all data are self-reported.

The survey consisted of 25 questions (see Supplementary file). Four questions collected data on participant demographics (age group, ethnicity, gender and the region/town of their usual healthcare provider). A further five questions asked background information about the participants diagnosis pathway: (i) who diagnosed their diabetes; (ii) how long ago they were diagnosed; (iii) type of diabetes; (iv) monitoring for pre-diabetes prior to diagnosis and (v) how they were diagnosed.

Five questions then asked about the information and resources that they were given to support their diagnosis including whether they felt that they were given enough information / resources by their primary healthcare team (e.g. GP or nurse) when they were first diagnosed to help understand their diabetes; who was the most useful in helping to understand and manage diabetes; iii) which sources of information had they been offered or accessed to find out more about diabetes; iv) referrals to diabetes support services; and v) understanding around medications.

Lastly, five questions were asked about current diabetes management including i) confidence managing insulin dose changes; ii) understanding medication changes with illness; iii) understanding of ‘HbA1c’; iv) changes in lifestyle and exercise since diagnosis and v) blood glucose monitoring. Participants were also asked to contribute to free-text answers about what they found most difficult about managing their T2D, whether they thought resources were culturally relevant to them, what was most difficult about managing their diabetes since diagnosis, advice to newly diagnosed diabetes patients / Healthcare providers (HCPs) about how and where to access information and whether there were specific medications questions that would be helpful for others to understand.

### Analysis

All survey data was downloaded from the online survey tool and coded in an excel spreadsheet. Responses were excluded from those participants who indicated that their diagnosis occurred more than three years ago and/or self-reported that they had type 1 diabetes (T1D). The proportion of participants selecting particular responses within a given question were compared using Chi squared tests, with significance accepted at P < 0.05. Where free-text responses were provided, these were broadly categorized and summarised only.

## Results

A total of 203 participants completed the survey, of which 18 indicated that their diabetes was diagnosed more than three years ago and seven reported that they had T1D. These were excluded, leaving 178 responses from newly diagnosed T2D patients for analysis.

Responses were collected primarily from the Waikato region, including Hamilton (n = 85) and regional Waikato (n = 27) but also from other parts of New Zealand including Northland (n = 2), Auckland (n = 15), Coromandel/Hauraki (n = 20), Bay of Plenty (n = 6), Wellington / Taranaki (n = 10) and Christchurch / Southland (n = 13).

Three quarters of participants who completed the survey were female, and nearly half of all respondents identified as Māori (41.0%) or Pacific (2.8%). Survey responses were well distributed across age groups, with two thirds of participants aged 45 years or older. The majority of patients were diagnosed by a GP in primary healthcare (71.5%) or at a Māori healthcare provider (15.2%), mostly via routine testing (e.g. known history of prediabetes) or patient presentation of symptoms. At the time of the survey 61.9% participants were managing their T2D with lifestyle modifications and/or non-insulin medications only, whilst the remainder required insulin to support glycaemic control. Participant demographics and diagnosis information are given in Table 1.

**Table 1:** Summary participant demographic characteristics (total n = 178)

Variable		Study population	
		*N*	%
**Age (years)**			
	18–24	6	3.4
	25–34	22	12.4
	35–44	35	19.7
	45–54	44	25.3
	55–64	44	24.7
	65–74	21	11.8
	> 75	6	3.4
**Ethnicity**			
	NZ European / European	77	43.3
	Māori	73	41.0
	Pacific	5	2.8
	Asian	12	6.7
	Other	11	6.2
**Gender**			
	Female	133	74.7
	Male	44	24.7
	Gender Diverse	1	0.6
**Who Diagnosed the Diabetes**			
	General practitioner	131	73.6
	Māori Health Provider	27	15.2
	Non-diabetes hospital visit	11	6.2
	Urgent care visit	1	0.6
	Other	8	4.5
**How was Diabetes Diagnosed**			
	Random test	38	21.3
	Routine diabetes monitoring	65	36.5
	GP – presentation with symptoms	50	28.1
	Non-diabetes hospital visit	17	9.6
	Community screening	0	0
	Did not answer	6	4.5

### Provision of diabetes resources

When asked if they felt if they were given enough information / resources by their primary healthcare team at the time of diagnosis to help understand their T2D, nearly three quarters of participants replied yes (n = 123; 71.1%; five no response), though nearly half of this group (n = 52) would have liked ‘more information’. In contrast, 50 participants (28.9%) responded ‘No’, that they did not receive adequate information or resources and that they ‘still did not really understand their diabetes’. The proportion of males and females in this latter group was comparable (28.4% vs. 31.0%) and there was no difference between ethnic groups (Māori (25.7%), NZ European (27.7%), Asian (33.3%), ‘Others’ (54.5%); P > 0.05). Participants responding ‘No’ were also distributed across practices throughout the different study regions.

**Fig. 1:**
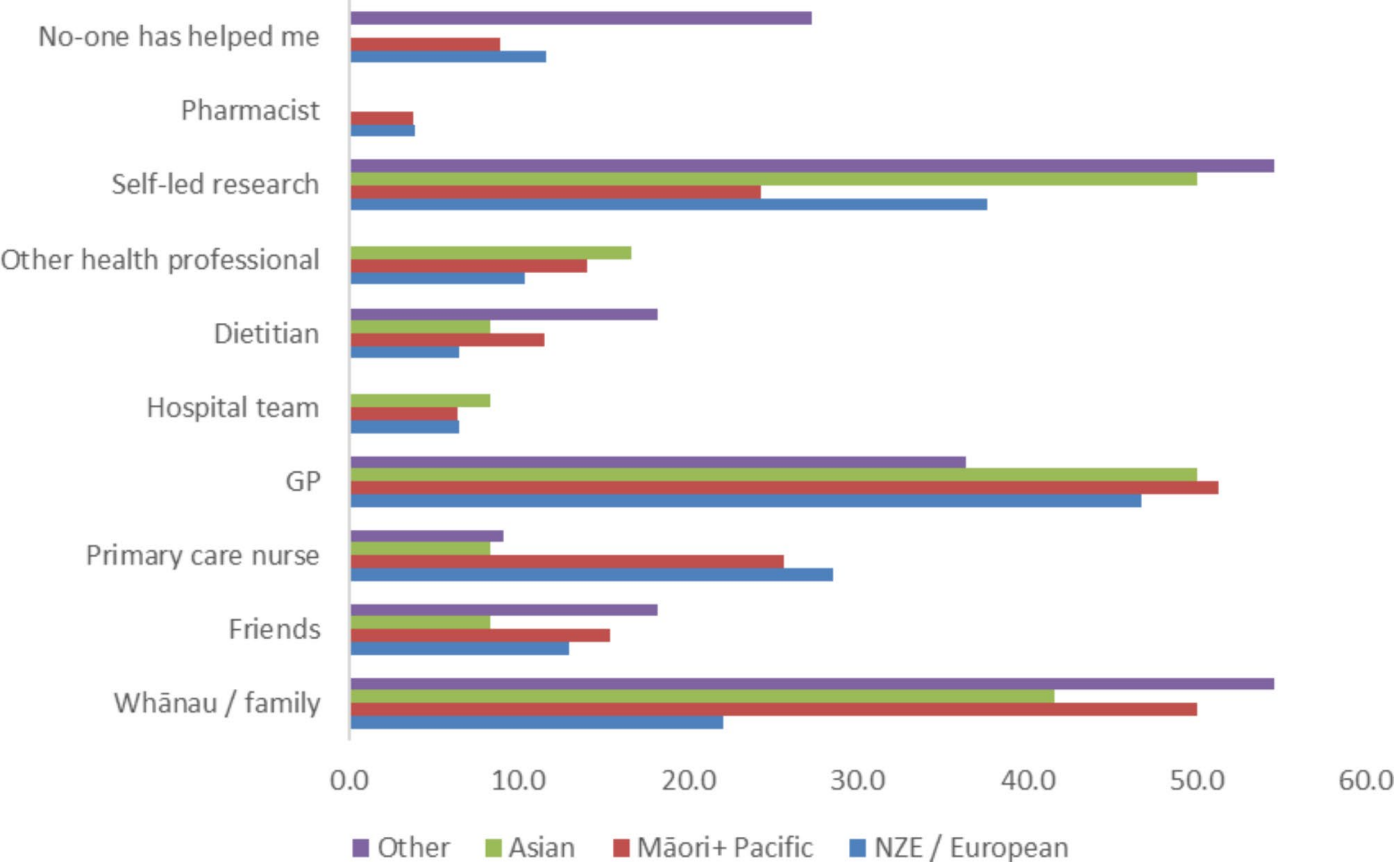
Resources that were self-reported to be the most useful in supporting the understanding of T2D after being newly diagnosed (percent)

The resources reported to be most useful in helping patients to understand and manage their diabetes are shown in Fig. 1. Whānau/ Family, the GP and self-led research were shown to be most useful, though Māori participants were less likely to undertake their own research than Asian and NZ Europeans (24.4% vs. 50.0% and 37.7%; P < 0.05).

Eighteen participants in total suggested that a dietician was helpful to understand their new T2D diagnosis (3.9 − 19.2%, Fig. 1), though these rates are higher when in including only those who indicated that they had received a diabetes referral (15.8, 25.0 and 31.2% of European, Māori/Pacific and Asian participants, respectively). Similarly, only six participants (three European and three Māori) indicated that a pharmacist had been a useful source of information, though this included 100% of those who had been referred to a pharmacist for follow-up support. Generally, referral rates to specialist services were low: dieticians (32.2%), diabetes specialist team (32.0%), pharmacist (19.5%), and lifestyle intervention or gym programs (16.2%).

At the time of the survey, the majority of patients (42.7% Yes and 31.5% Sometimes) felt that their HCPs were effectively supporting them to manage their T2D.

When we explored in more detail where newly diagnosed patients accessed diabetes resources, information was generally provided from HCPs, though one in five participants also sought to find information about lifestyle changes, specific Diabetes NZ resources and internet information themselves. HCPs were most likely to offer a diabetes-specific clinic appointment (64.5% of participants), information about medications (59.3%), lifestyle (diet/exercise) advice (51.2%), written information / pamphlets (53.0%) and/or Diabetes New Zealand resources (43.6%). Of concern, approximately a quarter of participants reported that they were never given any information/resources (28.4%) or a diabetes-specific healthcare appointment (27.4%) since being diagnosed.

### Understanding medications and blood test measurements

**Fig. 2:**
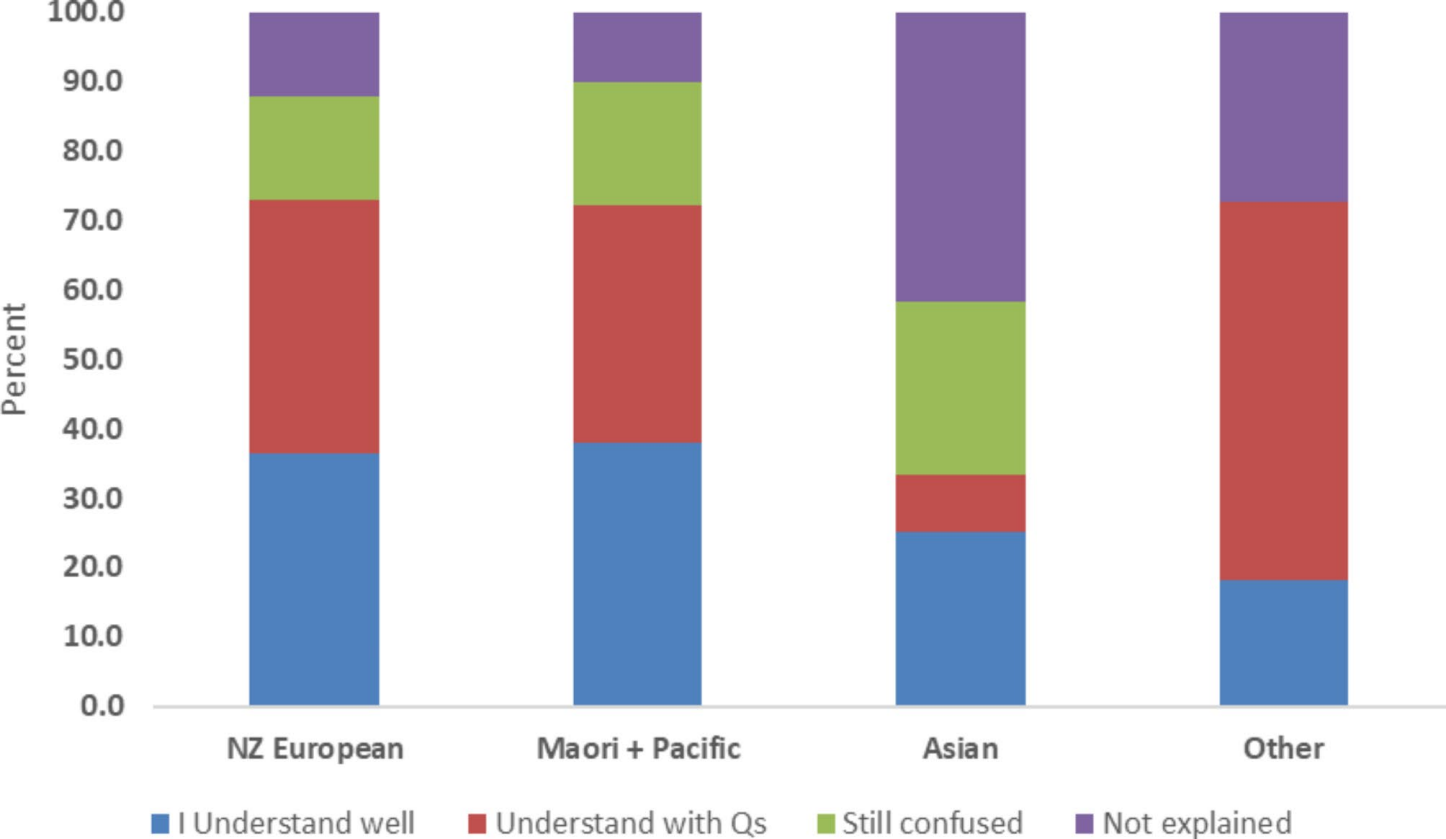
Proportion of patients, by ethnicity, who felt that medications were explained well to them, who had questions, were still confused or suggested that medications were not explained at all

As Fig. 2 shows, medication use was reported to be relatively well explained to two thirds of NZ European and Māori/Pacific participants, though at least half of these participants still had questions. However, only a third of Asian participants indicated that their medications had been adequately explained to them, with 40% of Asian respondents (5 of 12) indicating that these had not been explained at all (P < 0.05 vs. other ethnic groups). Overall, the proportion of patients who were confused about medications or felt they have not been explained did not differ by gender (29.3% of females vs. 29.5% of males; p = 0.92) or whether they had seen a pharmacist/pharmacist prescriber (14.3% vs. 22.3% of those who felt they had adequate explanation; P = 0.243). Similarly, the proportion of participants answering that they were ‘confused’ or that ‘medications were not explained’ did not differ by age group other than in those aged greater than 65 years (19.0% vs. 29.7–31.8% in other age groups, P < 0.01).

Of the 72 participants who indicated that they required insulin to manage their T2D, three quarters (75.8%) said that they were confident (‘yes’ or ‘somewhat’) in managing their insulin dosages as they altered food intake or other lifestyle factors. Those who said they struggled to understand this were more likely to be Māori than NZ European (25.0% vs 20.8%; of Māori and NZE insulin users P = 0.041). Overall, only 40.0% of participants knew how to alter their medication during times of illness.

A total of 65.2% of participants were fully or somewhat confident with understanding what an HbA1c measurement was, though a further 13.1% reported that they had heard of it but didn’t know what it meant. In addition, 21.7% of participants had either ‘not heard of it / had no idea’ what HbA1c referred to.

The majority of participants also recorded lifestyle changes in response to their T2D diagnosis: 71.0% reported a decrease in alcohol intake, 43.7% had an increase in home cooking and/or healthy foods and 39.3% reported an increase in exercise activity.

### Participant comments

In free-text comments, participants were asked to comment on what they found to be the most challenging aspect of managing their T2D. The most common answers (48 responses) were associated with diet and exercise, including knowing what they should and shouldn’t be eating, eating foods with fewer carbohydrates and sugar / avoiding sweet things and being able to afford ‘more healthy foods’. A further 28 responses commented on the challenges of taking medications, including managing with insulin (including around exercise) and the side effects associated with many agents.

Participants also provided comments on whether they found the resources given to them to be culturally relevant. Responses included the fact that written resources were often ‘too wordy’ and that it ‘would have been nice to have this [resource] in their own language’. One woman noted that ‘her doctor did not involve her existing whānau [family] when discussing food choices’, and another reiterated the need for ‘whakawhanaungatanga’ [connectiveness between patient and doctor] to allow her to discuss her diagnosis in a safe manner. Several participants commented that the written resources were difficult to understand and not relevant to them and their whānau/family.

Many participants provided free-text information as feedback to HCPs about interacting with patients with a new diabetes diagnosis. This included having more empathy about the changes they were expecting of their patients and ‘understanding their story’, using appropriate language that patients can understand, and to generally be ‘more kind, gentle and supportive’. Several also suggested that it would have been helpful to have been offered access to support groups (online and offline). Table 2 offers a sample of the range of comments from survey findings.

**Table 2:** Selection of patient comments

Q1 Advice you would give to newly diagnosed patients?	Q2 The most difficult part of managing diabetes since diagnosis?	Q3 Are there any questions you have about your diabetes or medications?	Q4 What advice would you give HCP for newly diagnosed?
*Seek all info until you feel confident to know what you have and how to treat diabetes and don’t settle for quick advice and pamphlet giving to move you along.(P 55)*	*Not being sure what is the difference in symptoms of ‘hypo’ or ‘hyper’ and what to do if you have them (P 34)*	*What is diabetes and how it actually works in the body. What is it lack of and how it can improve (P 32)*	*Take time and explain everything. Follow up at least a week or 2 after diagnosis so they can answer questions after the information sinks in, not at 3 months (P 13)*
*Take all the information and services available, research on your own also talk to your whānau (P 90)*	*Information about medications and diet. Carbs are in everything. How am I supposed to not eat these? What should I eat instead? (P 114)*	*I would love some guidance around how to manage my diet and what kind of glucose levels I should be aiming at if I was to be checking after every meal. (P 40)*	*Please be kind and non judgemental. It is a shock to get a diagnosis & this is completely life changing and overwhelming. Don’t leave your patient on their own to deal with it, please follow up and check on their mental state and support them. (P 21)*
*I dont know as I am still trying to find all of this myself.( P 19)*	*Understanding how to read my (glucose) levels. I have no idea (P 17)*	*Nope fully understand (P 118)*	*Realise there can be grief over not been able to eat the way they once could. That can be scary, and that suddenly everything has changed. (P 76)*
*That there are support groups out there that will give all the information you need to help you manage your diabetes (P 98)*	*Trying to cut or stop eating sweet stuff and breads that’s the hardest for me. Plus, healthy stuff is mor expensive (P 27)*	*My doctor said something adjusting my meds when I’m sick. To be honest it was all pretty overwhelming and I didn’t really take in what she was saying. So I don’t know.*	*Make sure your patient understands the disease and all its ramifications, and the medication. Also, the need for a good diet and exercise plan. Also, where to find support systems. (P 125)*

## Discussion

T2D is increasingly prevalent in New Zealand, but there appears to be much variation in how prepared patients feel about being able to self-manage their disease. Importantly, approximately two thirds of patients felt that they had been provided with adequate resources and education. This is encouraging, particularly given the fact that T2D management can vary dramatically between practices, and that clinical inertia and health-system barriers in New Zealand have all been shown to impact on access to care. Conversely, nearly a third of participants indicated that they had not been appropriately informed by their primary care team to manage their T2D, with many others relying on other sources of information to guide their management and understanding. This suggest that there is still a need in New Zealand to better support patients who are newly diagnosed with T2D given that this has been identified as being a key intervention timepoint to ensure optimal self-management and positive health outcomes [15, 16].

It is interesting to note that while T2D is predominantly diagnosed and managed in primary care, the role of the GP or nurse was only considered to be a useful source for information by approximately half of all participants. T2D and its management are considered to be complex [10] and it has been suggested that patient education offered in primary care may be limited by the shorter consultation times (usually only 15 min) and a lack of appropriate diabetes knowledge by generalist clinical staff [22]. However, all GPs should have a good foundational knowledge of T2D that they can share with their patients. While there is still variation nationally, many practices in New Zealand now offer additional services as a part of their multidisciplinary teams, such as specialist diabetes nurses and pharmacist prescribers. However, whilst primary care can refer patients on to additional services, specific services such as dietician and psychology support are generally rare and/or difficult to access. Other recent changes to health delivery have also included the use of mobile or telehealth rather than clinic-based staff with the aim of reaching and engaging with remote and often highly-deprived communities [27, 28]. The effects of these teams on patient-level and health system outcomes are yet to be evaluated.

Interestingly, many patients reported that they sourced their information about T2D and its management through friends and family/whānau, though the reliability of this information is questionable due to the subjective experiences of T2D and the presence of unregulated websites and social medial platforms offering ‘health’ information. Importantly, New Zealand does offer a range of diabetes websites and information from more legitimate sources, (e.g. NZ Diabetes website, the Ministry of Health, Te Whatu Ora (Health New Zealand) and Health Navigator), but many patients in this study indicated that the information found through self-research avenues was not relevant in a New Zealand context, particularly for Indigenous Māori and other minority groups. As such, recent interventions have been developed to address T2D within a cultural context, [29] and culturally-appropriate resources are being developed [30–32]. Such resources and programs need to be urgently expanded upon (including things like HbA1c, medications and sick day management) and made available to both patients and providers in the T2D community, including for community groups for whom English is not their first language.

Similarly, the literature also reports that the patient-clinician relationship is essential for maintaining ongoing care, and we suggest that this may have contributed to why many of our participants reported that they felt under-prepared to manage T2D. In New Zealand, it is well established that a Westernised model of healthcare creates barriers to access and ongoing care for Indigenous Māori, [33–35] and this must also be addressed to ensure both optimal and equitable access to timely T2D resources. In line with this, strong therapeutic relationships need to be established for as many patients as possible, working alongside patients in a ‘with them’ rather than a ’to them’ framework, being cognisant of cultural, spiritual and other needs. Many health providers in New Zealand are now meeting this need via the inclusion of multilingual clinical staff and the use of Māori health providers that follow a Te Ao Māori (Māori worldview) framework, and whilst their role in diabetes management has not been specifically reported on, the latter have been shown to significantly improve health access and outcomes for Māori [33, 36]. However, we do note that existing workforce pressures in healthcare in New Zealand also create barriers to these relationships as the extended wait times for appointments make it increasingly challenging to see the same GP or nurse for repeat visits. Regardless, we suggest that there is a strong need to evaluate whether the current T2D resources are culturally relevant and appropriate for use in Indigenous and migrant groups within New Zealand, and to codesign resources as appropriate to meet the needs of these population groups [36].

Whilst our study has yielded interesting information about the provision of resources and patient preparedness for managing T2D we do note a number of study limitations. Firstly, our study population was sourced from two different cohorts (patients enrolled with a regional primary healthcare provider, and secondly via social media), and it is probable that these two groups will have differed with regard to demographics. Due to the anonymous nature of the survey we are unable to determine which participant responses originated from which group, but this should be considered when interpreting the findings. In addition, both methods of recruitment relied on participants having a reasonable level of digital/technical ability to be able to access the survey, and it is possible that our participants may inherently be those who more readily access online educational resources. As such it would be valuable to repeat this survey using alternative, people-centric methodologies to ensure that the responses of those in harder to reach groups (e.g. socioeconomically-deprived and those with reduced health or digital literacy) are also captured.

We also note that our survey relied on self-reporting of variables such as ‘type of diabetes’ and knowledge of HbA1c and medication use, and due to the anonymous nature of this survey we were unable to validate these responses. Thus, it is possible that some participants may be misinformed or have underestimated their knowledge levels [37]. Similarly we have not evaluated any of the content that participants have reported accessing via different channels and it likely that at least some of what was sourced through online means and unregulated sources was useful. Third, we note that our participant cohort will not be fully representative of the newly diagnosed T2D community as a whole, particularly as access to primary care and education (for both patients and clinicians) varies across the country. Thus, we suggest that a review of these sources is needed to better understand what patients with T2D are routinely accessing throughout New Zealand alongside what specific materials and information are being provided by the primary healthcare team. Lastly, we note that multiple comments were provided in free-text responses and that this has only been briefly described here. Collectively, the aforementioned points all suggest the need for a larger study to better understand the nuances of accessing care for diabetes management, and how the early experiences at the time of diagnosis frame and shape a persons understanding of T2D management going forward.

Conclusion: New Zealand appears to be doing a relatively good job of preparing patients with newly diagnosed T2D to be able to self-manage their condition. However, there is still a need to address the gaps, including the quarter of participants who reported that they did not feel they were being supported or provided with appropriate resources. In particular, there may be a need for more culturally-specific materials (including for Māori, Pacific and/or Asian communities) as well as for more available time where patients can discuss their questions with their primary care team.

### Electronic supplementary material

Below is the link to the electronic supplementary material.

Supplementary Material 1

## Data Availability

The datasets used and/or analysed during the current study are available from the corresponding author on reasonable request.

## Abbreviations

T2D: Type 2 diabetes
GP: General practitioner
HCP: Healthcare provider
T1D: Type 1 diabetes
