# Supplementary material for: Newly diagnosed type 2 diabetes – does New Zealand General Practice adequately prepare patients to self-manage their Condition?
Source: BMC Prim Care. 2023 Aug 21;24:162. doi: 10.1186/s12875-023-02118-1 (PMC10441743; doi:10.1186/s12875-023-02118-1)
Supplement: Supplementary file 1 — Supplementary Material 1 [file 12875_2023_2118_MOESM1_ESM.pdf]

Exploring resources for adults diagnosed with type two diabetes in Aotearoa

Nau mai, haere mai! Welcome, and thank you for participating in this survey

Te Huataki Waiora, School of Health at the University of Waikato are wanting to improve the experiences of patients, whānau and family living with type 2 diabetes.

Kaupapa, about this project:

In New Zealand there appears to be much variability in the information and resources that are provided to patients with newly diagnosed Type 2 Diabetes. We are keen to hear from patients who have been recently diagnosed about your experience of being diagnosed, and what support, information and resources were given to you. We invite you to complete the short survey below. This should take no more than 10 minutes.

Your involvement is completely optional and anonymous, and no information about you has been shared with the research team. If you have any questions about this study please contact the project lead, Associate Professor Lynne Chepulis at: lynnec@waikato.ac.nz

Ngā mihi ki a koutou, thank you for taking the time to complete this survey.

1. Please tell us a little bit about yourself - how old are you?

- ◯ Under 18
- ◯ 18-24
- ◯ 25-34
- ◯ 35-44
- ◯ 45-54
- ◯ 55-64
- ◯ 65-74
- ◯ > 75 years

2. What gender do you identify with?

- ◯ Female
- ◯ Male
- ◯ Gender Diverse

3. What ethnicity do you identify with?

- ◯ Māori/Tangata Whenua
- ◯ NZ European/Pākehā
- ◯ Pasifica
- ◯ Asian
- ◯ Other, please specify

4. What region, or town is your usual GP located?

5. Which of the following health providers best represents provider who diagnosed your diabetes?

- ◯ Māori primary healthcare provider
- ◯ GP (primary healthcare)
- ◯ Urgent care (e.g after hours clinic that you don't visit for routine care)
- ◯ Public hospital
- ◯ Other

6. How would you rate your relationship with your regular health care provider?

0 - Terrible 50 - Okay 100 - Perfect

7. Approximately how long ago were you diagnosed with diabetes?

- ◯ 0-12 months
- ◯ 1 year
- ◯ 2 years
- ◯ 3 years
- ◯ 4 years
- ◯ 5+ years

8. What type of diabetes do you have?

- ◯ Type two - diet controlled or oral medication (pills) - e.g metformin
- ◯ Type two - now managed using insulin after using oral diabetes medications
- ◯ Type 1 - managed with insulin from the first day of diagnosis
- ◯ Not sure

Comments

9. Were you being monitored for pre-diabetes before being diagnosed with type two diabetes?

- ◯ Yes
- ◯ No

10. Do you feel that you were given enough information / resources by your primary healthcare team (eg GP or nurse) when you were first diagnosed to help you understand your diabetes?

- ◯ Yes, I think I know all that I need to
- ◯ Yes, though I would have liked more information
- ◯ No, I still don't really understand my diabetes

Comments

11. Who do you find is the most useful in helping you to understand and manage your diabetes? (select all that apply)

- ☐ Whanau / family
- ☐ Friends
- ☐ primary care nurse
- ☐ GP (primary care doctor)
- ☐ Hospital team
- ☐ Dietitian
- ☐ Other healthcare professional
- ☐ myself - I research online
- ☐ Pharmacist
- ☐ No-one has helped me to understand my diabetes
- ☐ Other

12. How was your diabetes diagnosed?

- ◯ GP - routine monitoring
- ◯ GP - random test
- ◯ GP - you presented with symptoms and asked for a check
- ◯ Hospital (e.g if there for another reason / condition)
- ◯ Hauora or community event - community screening

Comments

13. To date, which sources of information have you been offered or accessed yourself to find out more about diabetes?

	Not Provided	Searched for it myself	Offered to me by healthcare	Offered to me from family/friends
Diabetes NZ resources	○	○	○	○
Pamphlets	○	○	○	○
A session with a primary care nurse	○	○	○	○
A diabetes specific appointment with your healthcare provider	○	○	○	○
A session with a diabetes specialist	○	○	○	○
Lifestyle (diet and exercise) information	○	○	○	○
Information about medications	○	○	○	○
Diabetes-specific Internet sources	○	○	○	○
Diabetes self-management group	○	○	○	○

Please add any additional resources here

____

14. Were you ever referred to any of the following services to support you with your diabetes management?

	Yes	No
Dietitian	○	○
Lifestyle intervention program	○	○
Gymnasium	○	○
Diabetes specialist team	○	○
Diabetes support program	○	○
Pharmacist	○	○
Other	○	○

details:

____

15. When you were diagnosed, how well did you feel medications were explained to you?

- ◯ Very well, I had no questions
- ◯ Okay, I understand but I still have a few questions
- ◯ I'm still confused about medications (type of medication, doses, why I'm taking it etc)
- ◯ Not explained at all, I dont understand how my medications work

16. If you need to take insulin, do you feel confident in managing your insulin dosages as you alter your food intake, or other lifestyle factors?

- ◯ Yes
- ◯ Somewhat
- ◯ No, I struggle
- ◯ No, I cant
- ◯ I dont take insulin

17. Do you know what to do with your medication usage when you get sick?

- ◯ No
- ◯ Yes
- ◯ Comments

18. HbA1C, known as 'glycated haemoglobin', is a blood test that gives health care providers a measure of your blood sugar levels over the last three months.
Do you know what your 'HbA1c' is and what this explanation above means?

- ◯ Yes, I am confident with my understanding of HbA1C
- ◯ Yes, somewhat confident - I know there's more for me to learn
- ◯ I've heard the term 'HbA1c' but don't understand it
- ◯ I have no idea what that means / never heard of it
- ◯ no answer

Please note your HbA1c here if known

19. Since being diagnosed with diabetes have you changed any of the following?

	I do less	I do the same	I do more	Not sure
Exercise	◯	◯	◯	◯
Alcohol intake	◯	◯	◯	◯
Healthy eating	◯	◯	◯	◯
Home cooking	◯	◯	◯	◯
Smoking	◯	◯	◯	◯

20. Do you regularly monitor you blood glucose (sugar) levels by fingerprick or glucose monitor?

- ◯ Yes, before each meal
- ◯ Once daily
- ◯ Weekly check at home
- ◯ During routine GP checks only
- ◯ I don't need to check my blood sugar level regularly
- ◯ I prefer to not check my blood sugar levels
- ◯ Other, please specify

21. Do you **NOW** feel supported by your healthcare professionals to manage your diabetes

- ◯ Yes
- ◯ No
- ◯ Sometimes
- ◯ Other (please specify)

22. What thoughts / suggestions do you have about how culturally-relevant or appropriate any provided resources were for you?

23. What has been the most difficult part about managing your diabetes since you were diagnosed?

24. What advice would you offer (if any) to **people who have been newly diagnosed** with diabetes about how and where to access information?

25. What advice would you offer (if any) to **healthcare providers** who are working with people who have been newly diagnosed with diabetes about how and where to access information?

26. Are there any questions that you have about your medication or the diabetes disease that would be helpful to understand?

27. Would you be interested to speak more about your experiences with Type 2 Diabetes and information you have received, or ways that healthcare can be improved? If so, we would like to hear from you.

We are conducting a second part to this study where we look in more depth at peoples experiences and identify ways that healthcare can be improved for those newly diagnosed with diabetes in the future. If you would like to share more about your experience please contact us for a confidential 30-60 minute interview where you will be compensated for your time. Please note that if you select YES here and provide contact details, these will be passed onto a researcher at the University of Waikato to conduct this interview.

- ○ No, thank you
- ○ Yes, please provide a contact email address or phone number

Contact info:

28. I consent to my results being anonymously shared with the research team

- ☐ Yes
- ☐ No

29. Thank you for participating in this survey. To receive a $10 petrol voucher as a thank you from us, please provide a postal address.

- ○ No Thank you
- ○ Address:
